# Interleukin 17A Promotes Hepatocellular Carcinoma Metastasis via NF-kB Induced Matrix Metalloproteinases 2 and 9 Expression

**DOI:** 10.1371/journal.pone.0021816

**Published:** 2011-07-07

**Authors:** Jian Li, George Ka-Kit Lau, Leilei Chen, Sui-sui Dong, Hui-Yao Lan, Xiao-Ru Huang, Yan Li, John M. Luk, Yun-Fei Yuan, Xin-yuan Guan

**Affiliations:** 1 Department of Clinical Oncology, Li Ka Shing Faculty of Medicine, The University of Hong Kong, Hong Kong, China; 2 Department of Medicine, Li Ka Shing Faculty of Medicine, The University of Hong Kong, Hong Kong, China; 3 State Key Laboratory for Liver Research, Li Ka Shing Faculty of Medicine, The University of Hong Kong, Hong Kong, China; 4 Department of Medicine and Therapeutics and Li Ka Shing Institute of Health Sciences, The University of Hong Kong, Hong Kong China; 5 Department of Pharmacology and Department of Surgery, Cancer Science Institute, Yong Loo Lin School of Medicine, National University of Singapore, Singapore, Singapore; 6 State Key Laboratory of Oncology in Southern China, Sun Yat-sen University Cancer Center, Guangzhou, China; University of Hong Kong, Hong Kong

## Abstract

**Background:**

IL-17A is a pro-inflammatory cytokine that plays important role in inflammatory disease pathology and tumor microenvironment. The aim of this study is to investigate the effect of IL-17A on the progression of hepatocellular carcinoma (HCC).

**Methodology and Principal Finding:**

Expression pattern of IL-17A in clinical HCC samples (n = 43) was determined by immunohistochemistry staining. Transcript levels of MMP2, MMP9 and IL-17A were measured in another 50 pairs (including tumor and related non-tumor tissues) HCC samples. Cell growth, focus formation, cell migration, invasion and western blot assays were used to characterize the functional and signaling mechanisms in IL-17A-treated HCC. Association study was used to identify clinical significance of IL-17A in HCC. Compared with paired non-tumor tissue, higher frequency of IL-17A-positive cells was detected in tumor tissues in HCCs with metastasis, and the frequency of IL-17A-positive cells was also significantly associated with poor prognosis of HCC (*P* = 0.01). Functional study found that IL-17A could promote HCC cell migration and invasion. Further molecular analysis also showed that IL-17A could upregulate MMP2 and MMP9 expression via NF-κB signaling activation.

**Conclusions:**

IL-17A could promote HCC metastasis by the upregulation of MMP2 and MMP9 expression via activating NF-κB signaling pathway.

## Introduction

Hepatocellular carcinoma (HCC) is the fifth most common cancer word wide and it is also one of the poorest prognosis tumors in the world [1]. HCC often develops from chronic liver inflammation environment where plenty of leukocytes infiltrate [1], [2]. Recent studies find that immune cells and their secreted cytokines can not only contribute to the elimination of cancer cells, they could also provide a proper microenvironment for tumor development as well as promote tumor progression [3], [4], which is determined by the local tumor microenviroment and the function state of immune cells. For example, IFN-γ producing Th1 and CD8+ cytoxtic cells are associated with good prognosis [5], while interleukin 10 (IL-10) and TGF-β producing regulatory T cells are associated with poor prognosis in HCC [6], [7]. Another interesting example is the effects of macrophages on tumor development, which mainly depends on whether they secrete anti-tumor factors IL-12 and TNF-α, or pro-tumor factors IL-10, VEGF, PDGF, CXCL8, MMP-9 and TGF-β [4], [8].

Interleukin 17A (IL-17A) is a pro-inflammatory cytokine secreted by helper T cells (Th17), CD8 positive T cells, neutrophils, gamma/delta T cells and NK cells [9]–[12]. IL-17A has been found to play important role in many chronic diseases such as rheumatoid arthritis [13], inflammatory bowel disease [14] and multiple sclerosis [15]. Recently IL-17A has been also frequently detected in many cancers such as ovarian cancer [16], breast cancer [17] and gastric cancer [18]. The role of IL-17A in the development and progression of cancer remains controversial. Using animal model, some studies find that IL-17A can inhibit tumor growth and metastasis through IFN-γ producing NK and T cells [19], [20]. While other studies show that IL-17A can promote tumor growth and metastasis through IL-6/Stat3 signaling pathway [21] or through the induction of tumor promoting microenvironment at tumor site [22].

In the present study, we found that IL-17A was frequently overexpressed in HCC with metastasis. The frequency of IL-17A-positive cells in tumor tissue was associated with HCC metastasis and prognosis. Further study found that IL-17A could increase cell motility by the upregulation of matrix metalloproteinases 2 (MMP2) and 9 (MMP9) via activating nuclear factor-κB (NF-κB) transcript factor.

## Results

### IL-17A-positive cells were associated with HCC metastasis

**Figure 1:**
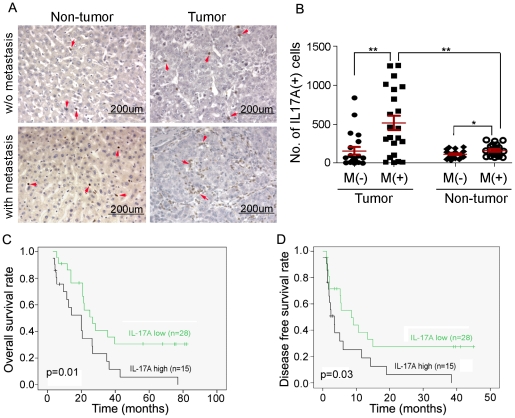
Expression and prognosis of IL-17A in HCC. (**A**), Representatives of IL-17A expression in primary HCCs (tumor vs non-tumor tissues) with or without metastasis detected by IHC (magnification 200×). Positive IL-17A staining cells are indicated by red arrows. (**B**), Compared with HCC without metastasis (M-), the frequency of IL-17A positive cells was significantly higher in both tumor and non-tumor tissues in HCC with metastasis (M+). The frequency of IL-17A positive cells in tumor tissue was significantly higher than that in non-tumor tissue in HCCs with metastasis (M+). **, *P*<0.01; *, *P*<0.05. (**C**) and (**D**), Kaplan-Meier survival analysis showed that HCC patients with higher density of IL-17A positive cells had lower overall survival (**C**) and disease free survival rates (**D**).

The number and distribution of IL-17A-positive cells were compared by IHC staining between primary HCC specimens with and without metastasis. IL-17A-positive cells could be detected in both tumor and adjacent non-tumorous tissues (Figure 1A). In 21 HCC cases without metastasis, no significant difference (*P* = 0.391, paired-samples T test) was observed in the frequency of IL-17A-positive cells between tumor (mean: 157±98 cells, in 10 continuous fields under 400× microscopy) and adjacent non-tumorous tissues (mean: 114±22) (Figure 1B). In 22 HCC cases with metastasis, the frequency of IL-17A-positive cells was significantly higher (*P* = 0.001, paired-samples T test) in tumor tissue (mean: 516±182) than that in adjacent non-tumorous tissues (mean: 164±31, Figure 1B). The frequency of IL-17A-positive cells in tumor tissues was significantly higher in HCC cases with metastasis than that without metastasis (*P* = 0.002, Independent sample T test). Interestingly, the frequency of IL-17A-positive cells in adjacent non-tumorous tissue was also higher in HCC cases with metastasis than that without metastasis (*P* = 0.013, Independent sample T test) (Figure 1B).

### IL-17A-positive cells were associated with poor prognosis of HCC

To investigate the correlation of the frequency of IL-17A-positive cells in tumor tissue with clinic pathological features, the mean of IL-17A-positive cells in tumor tissue in 43 HCC cases was calculated. According to whether the frequency of IL-17A-positive cells was above the mean level (341 cells) in tumor tissue or not, HCC cases in the present study were divided into two groups: IL-17A-high group (above the mean level, n = 15) and IL-17A-low group (below the mean level, n = 28). The association study showed that the frequency of IL-17A-positive cells in tumor tissue was not significantly associated with patient's gender, age, HBV infection, cirrhosis, tumor size and TNM stage (Table 1). Interestingly, high frequency of IL-17A-positive cells in tumor tissue was significantly associated with patient's metastasis (*P* = 0.002, Table 1), overall survival rate (*P* = 0.01, Figure 1C) and disease-free survival rate (*P* = 0.03, Figure 1D). Univatiate and multivariate Cox progression analysis were performed and the results showed that the frequency of IL-17A-positive cells in tumor tissue was an independent prognostic factor for overall survival (HR = 0.236, P = 0.001) and disease-free survival (HR = 0.444, P = 0.027) (Table 2).

**Table 1:** Correlation of IL-17A(+) cells with clinicopathological features in 43 HCC patients.

Clinicopathological Features		Number	IL-17A(+) cells in tumor (Mean±2SED)	*P*-value
***Gender*** *	Female	6	427±373	.589
	Male	35	314±130	
***Age***	≤60	33	339±143	0.744
	>60	8	296±217	
***HBsAg***	Negative	5	336±340	.980
	Positive	38	341±127	
***Cirrhosis***	Absent	5	389±451	.837
	Present	38	334±119	
***Serum AFP (ng/ml)*** *	≤25	17	293±171	.440
	>25	25	385±165	
***Tumor size (cm)***	≤5	6	396±382	762
	>5	37	332±123	
***Tumor multiplicity***	Solitary	27	392±147	.263
	Multiple	16	254±192	
***TNM stage***	I–II	25	268±122	.179
	III–IV	18	441±219	
***Metastasis***	No	21	157±98	**.002**
	Yes	22	516±182	

*Partial data unavailable, statistics was done on the available data. Difference is considered significant when *P*<0.05 (shown in bold). HBsAg: Hepatitis B surface antigen; AFP, a-fetoprotein.

**Table 2:** Univariate and multivariate analyses of variables associated with survival.

Variables	Overall Survival				Disease Free Survival			
	Univariate	Multivariate			Univariate	Multivariate		
	P value	Hazard Ratio	95% CI	P value	P value	Hazard Ratio	95% CI	P value
***Gender*** * ** :** male vs female	.085			NA	.247			NA
***Age*** *:* >60 vs ≤60	.220			NA	.346			NA
***HBsAg:*** positive vs negative	.790			NA	.981			NA
***Cirrhosis:*** present vs absent	.148			NA	.060			NA
***Serum AFP *** * ***:*** >25 vs ≤25 ng/mL	.229			NA	.089			NA
***Tumor size:*** >5 vs ≤5 cm	.471			NA	.260			NA
***Tumor number *** *:* multiple vs solitary	.129			NA	.225			NA
***TNM stage:*** III–IV vs I–II	**.004**	5.875	2.477–13.933	**.000**	**.039**	2.412	1.122–5.187	**.024**
***Vascular invasion:*** present vs absent	**.015**	3.745	1.679–8.356	**.001**	**.038**	2.014	0.968–4.189	.061
***Intratumor IL-17A positive cells frequency:*** high vs low	**.005**	0.263	0.123–0.564	**.001**	**.014**	0.444	0.217–0.910	**.027**

*Partial data unavailable, statistics was done on the available data. Difference is considered significant when *P*<0.05 (shown in bold). NA, not adopted.

### Recombinant human IL-17A (rhIL-17A) could not promote HCC cell growth in vitro

**Figure 2:**
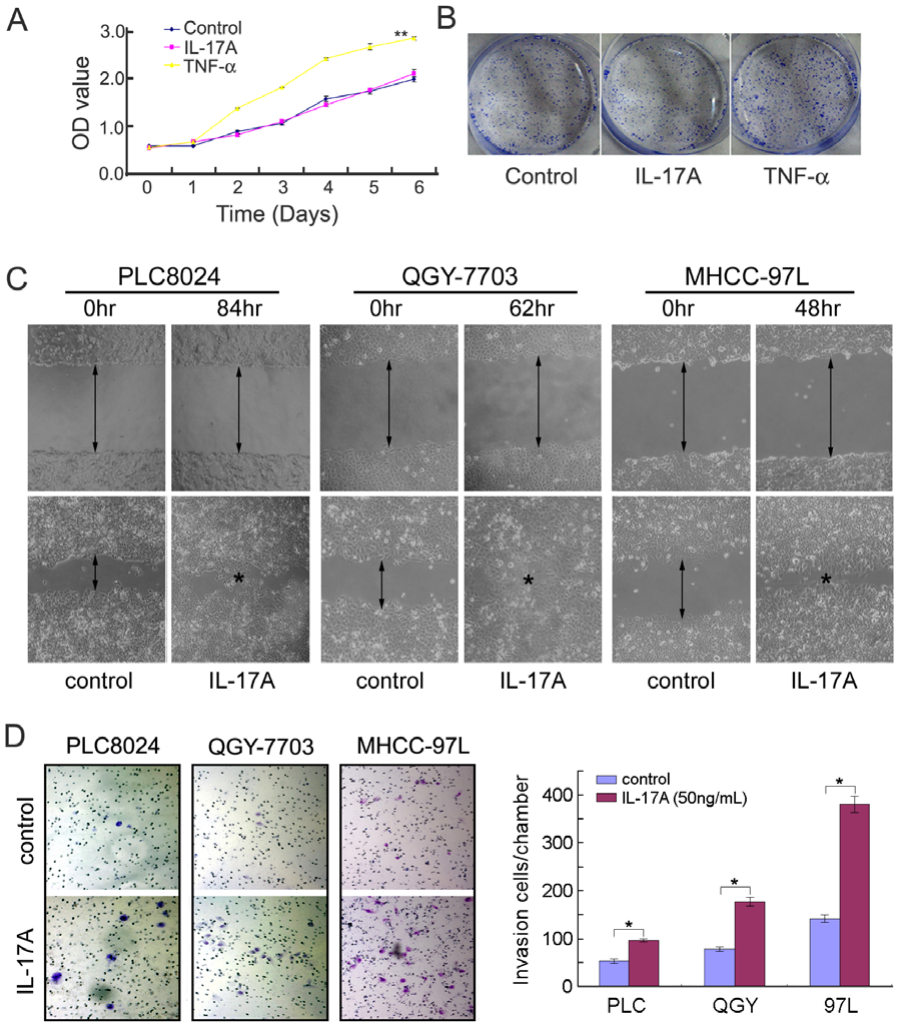
RhIL-17A promoted HCC cell migration and invasion. (**A**), Cell growth rate between PLC8024 cells treated with and without rhIL-17A (50 ng/mL) or TNF-α (10 ng/mL) was compared by XTT assay.**, P<0.01. (**B**), Representatives of foci formation in monolayer culture of PLC8024 cells treated with or without rhIL-17A (50 ng/mL) or TNF-α (10 ng/mL) for a week. (**C**), RhIL-17A treated HCC cells (PLC8024, QGY-7703 and MHCC-97L) showed higher motility in a wound-healing assay, compared with cells without RhIL-17A treatment. (**D**), Effect of RhIL-17A on cell invasion was detected by cell invasive assay. Representatives of cells migrated through Matrigel-coated transwell were shown in the left panel (magnification 100). Total invasive cell number in each chamber was summarized in the right panel. *, *P*<0.05.

Cell growth assay and cell proliferation assay were used to study the effect of rhIL-17A on cell growth in PLC8024 cells [23] by treating the culture cells with rhIL-17A. Cell growth rate was tested by XTT assay and the result showed that the cell growth rate was similar between cells treated with and without rhIL-17A (Figure 2A). Foci formation assay also showed that rhIL-17A could not increase foci formation ability in PLC8024 cells (Figure 2B).

### RhIL-17A increased cell motility in HCC cells

The effect of IL-17A on cell motility was investigated by wound healing and matrigel invasion assays. The wound healing assay showed that rhIL-17A could remarkably promote cell migration rate at the edge of exposed regions in PLC8024, QGY-7703 [24] and MHCC-97L [25] cells, compared to control parental cells (Figure 2C). Moreover, the invasion assay showed that the invasiveness of rhIL-17A treated cells was significantly higher than control parental cells (*P*<0.05, Independent Student's *t*-test) in all three tested cell lines (Figure 2D). These data demonstrated that IL-17A could enhance HCC cell migration and invasive ability.

### RhIL-17A could not promote the epithelial-mesenchymal transition (EMT) in HCC cells

**Figure 3:**
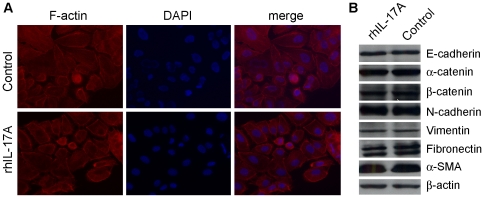
RhIL-17A did not affect cell cytoskeleton and EMT. (**A**), IF was used to detect F-actin distribution in PLC8024 cells treated with or without rhIL-17A (50 ng/mL) for 48 hr. (**B**), Western blot analysis was used to compare expression levels of epithelial markers (E-cadherin, α-catenin and β-catenin) and mesenchymal markers (fibronectin, Vimentin, N-cadherin and α-smooth muscle actin) in PLC8024 cells treated with or without rhIL-17A (50 ng/mL) for 48 hr. β–actin was used as a loading control.

Changing in cell cytoskeleton and obtaining cell motility through EMT is one of the features of metastasis cells [26], [27]. The effect of IL-17A on EMT was tested in PLC8024 cells. After treatment with 50 ng/ml rhIL-17A for 2 days, F-actin in PLC8024 was detected by immunofluorescent staining with rhodamine phalloidine. The result showed that no obvious change in F-actin distribution was detected between rhIL-17A treated and untreated control cells (Figure 3A). As EMT is one of the important mechanisms for cell migration and invasion, we next studied the effect of IL-17A on EMT in PLC8024 cells. Both epithelia markers (E-cadherin, α-catenin and β-catenin) and mesenchymal makers (N-cadherin, Vimentin, Fibronectin and α-SMA) were compared by western blot analysis between rhIL-17A treated and untreated cells. The results showed that no obvious difference was detected between rhIL-17A treated and untreated cells for all tested markers (Figure 3B).

### RhIL-17A upregulated MMP2 and MMP9 expressions in HCC cells

**Figure 4:**
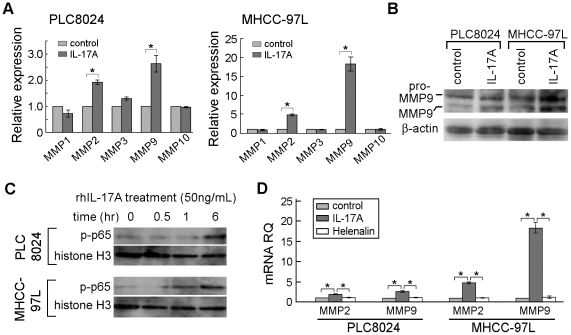
RhIL-17A upregulated MMPs expression via activating NF-κB. (**A**), Expressions of MMPs were compared by qPCR between cells treated with and without rhIL-17A (50 ng/mL) for 12 hours. *, *P*<0.05. (**B**), Expression of MMP9 was detected by western blot analysis in HCC cells treated with or without rhIL-17A (50 ng/mL) for 24 hours. (**C**), Western blot analysis was used to detect nuclear P-P65 (active form of NF-κB**)** expression in PLC8024 and MHCC-97L cells treated with rhIL-17A (50 ng/mL) at indicated time points. (**D**), Expressions of MMP2 and MMP9 were detected by qPCR in PLC8024 and MHCC-97L cells with different treatment. Control: without rhIL-17A treatment; IL-17A: treated with rhIL-17A (50 ng/mL); Helenalin: treated with helenalin (0.l µM) and rhIL-17A (50 ng/mL). *, *P*<0.05.

Since overexpression of MMPs plays an important role in cancer metastasis [28], we next investigated the role of IL-17A on MMPs expression in HCC cell lines. Expressions of MMP1, MMP2, MMP3, MMP9 and MMP10 were compared by qPCR between rhIL-17A treated and untreated cells. The result showed that MMP2 and MMP9 were upregulated in rhIL-17A treated cells (Figure 4A). As MMP9 has been reported to be an important factor in tumor metastasis [29], MMP9 was further characterized in this study. Western blot analysis found that MMP9 expression was upregulated in rhIL-17A treated cells (PLC8024 and MHCC-97L) compared to control (Figure 4B). It suggested that the metastasis promoting function of IL-17A might be through the extracellular matrix remodeling.

### RhIL-17A upregulated MMP2 and MMP9 expression via activating NF-κB

NF-κB has been reported as a downstream target of IL-17A signaling pathway in many cells [30], [31], which is able to upregulate MMP2 and MMP9 expressions [32]. And IL-17A was also reported to increase the expression of MMPs via activating NF-κB pathway in many cells [33], [34]. So We next tested whether the upregulating effect of IL-17A on MMP2 and MMP9 expressions in HCC cells was also via the activation of NF-κB or not. The result showed that the level of the active form of NF-κB (P-P65) in nuclei was dramatically elevated in PLC8024 and MHCC-97L cells after rhIL-17A treatment (Figure 4C). When helenalin, a NF-κB inhibitor, was added to PLC8024 and MHCC-97L medium before rhIL-17A treatment, MMP2 and MMP9 mRNA expression was significantly decreased (*P*<0.05, Independent Student's *t*-test) (Figure 4D). The result demonstrated that IL-17A induced MMP2 and MMP9 expression in HCC cells was via NF-κB activation. Accordingly, rhIL-17A induced HCC cell mobility could also be blocked by helenalin in vitro (Figure S1).

### IL-17A was positively correlated with expression of MMP2 and MMP9 in clinical samples

**Figure 5:**
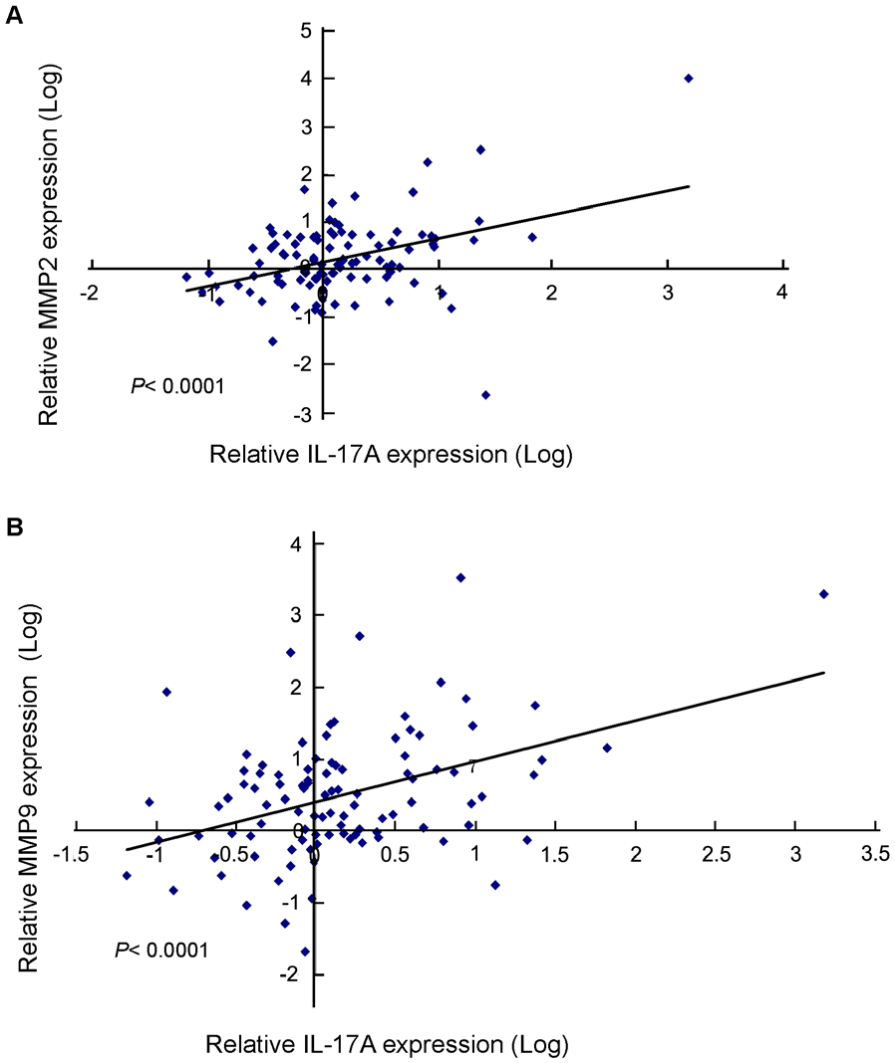
IL-17A correlated with MMP2 and MMP9 expression in HCC clinical samples. Expression of IL-17A was positively associated with MMP2 (**A**) and MMP9 (**B**) expressions in 50 clinical HCC specimens. Analyzed with linear regression lines and pearson correlation by SPSS16.0.

To confirm whether expression of MMP2 and MMP9 were correlated with IL-17A in clinical HCC samples, qPCR was used to detect expression of MMP2, MMP9 and IL-17A in 50 pairs (including tumor and related non-tumor tissues) HCC samples. The correlation study was then applied to analyze the qPCR data with SPSS16 software. The result found that expression of IL-17A was significantly correlated with expression of MMP2 (R = 0.998, *P*<0.0001) and MMP9 (R = 0.494, *P*<0.0001) in clinical HCC samples (Figure 5).

## Discussion

HCC is one of the most fatal diseases in the world because of its high recurrence rate after curative therapy and lack of biomarkers for early detection [35]. HCC mainly develops from chronic inflammatory disease [36], [37], where plenty of inflammatory cytokines infiltrate. IL-17A is an important inflammatory cytokines in the development of many inflammatory diseases and it is also frequently detected in tumor microenvironment [13]–[18]. Several recent studies show that Th17 and IL-17A expression CD8 T cells were attracted to and expanded by the local environment of HCC [38], [39] and increased intratumoral IL-17-producing cells were correlated with poor survival in HCC patients [37], while little is known about the effect of IL-17A on HCC progression. Recently, the effect of IL-17A on cancer progression has been addressed and results were controversial. Some reports showed that IL-17A could inhibit tumor growth and metastasis via the activation of cytotoxic T cells or inducing INF-gamma expression [19], [20]. While other studies demonstrated that IL-17A could promote cancer metastasis via affecting tumor microenvironment [21], [22] or inducing expression of other cytokines [40], [41].

In the present study, higher frequency of IL-17A positive cells in tumor tissue was significantly associated with poorer prognosis of HCC (*P* = 0.01) by promoting HCC metastasis, suggesting that IL-17A played an important role in promoting HCC progression. Functional study showed that IL-17A could enhance the migration and invasion abilities of HCC tumor cells. And IL-17A was also proved to can promote the motility of breast cancer cells in another study [42]. Snail induced EMT [43] and twist mediated morphology change [44] are the most important events in tumor invasion and metastasis, which involves the losses of cell adhesion, cell-cell tight junction, cell polarity and remodeling of the cytoskeleton so as to facilitate cell migration and invasion. So we first studied the effect of IL-17A on EMT progression, and the result found that IL-17A did not affect HCC cell morphology and EMT progression.

Since overexpression of MMP is another key factor for tumor invasion and metastasis [45], we next investigated whether IL-17A can affect MMPs expression. The result showed that IL-17A could upregulate expressions of MPP2 and MMP9. Overexpression of MMP2 and MMP9 has been frequently detected in solid tumors and associated with tumor invasion and metastasis [28], [29], including HCC [46], [47]. Therefore, the pro-metastasis effect of IL-17A on HCC might be through the upregulation of MMP2 and MMP9. qPCR result further demonstrated that expression of MMP2 and MMP9 were significantly (*P*<0.0001) correlated with IL-17A expression in clinical HCC specimens. As NF-κB is a key transcription factor in the regulation of MMP9 expression [32] and IL-17A has been reported to be able to activate NF-κB signaling [30], [31], we next studied whether IL-17A could activate NF-κB signaling pathway. The result found that rhIL-17A could activate NF-κB and subsequently upregulate MMP2 and MMP9 expression. This effect could be effectively inhibited by NF-κB inhibitor, suggesting that the upregulating role of IL-17A in MMP2 and MMP9 expression might be through the activation of NF-κB. Further characterization of the effect of IL-17A on HCC invasion and metastasis may lead to the identification of new diagnostic markers and therapeutic targets.

## Materials and Methods

### HCC specimens and cell lines

Forty-three HCC specimens from archives of paraffin embedded tissues were collected at the Sun Yat-sen University Cancer Center (Guangzhou, China). Among them, 22 HCCs with metastasis including 8 portal vein metastases, 9 intra-hepatic metastasis and 5 extra-hepatic metastases (4 in lung and 1 in centrum). Another 50 pairs of frozen HCC specimens (tumor and adjacent non-tumorous tissues) were collected at the Sun Yat-sen University Cancer Center (Guangzhou, China) for RNA isolation. Samples used in this study were reviewed and approved by the Committees for Ethical Review of Research involving Human Subjects at Sun Yat-Sen University Cancer Center. Human HCC cell lines QGY-7703 and PLC8024 were obtained from the Institute of Virology of the Chinese Academy of Medical Sciences (Beijing, China). MHCC-97L was obtained from Liver Cancer Institute, Fudan University (Shanghai, China). All cell lines were cultured in high-glucose DMEM (Gibco BRL, Grand Island, NY) supplemented with 10% fetal bovine serum.

### Immunohistochemistry (IHC) Staining

Paraffin-embedded, formalin fixed liver tissue sections (5 µm in thick) were deparaffinized and rehydrated. The endogenous peroxidase activity was blocked with 3% hydrogen peroxide (H_2_O_2_) for 30 min. For antigen retrieval, slides were immersed in 10 mM citrate buffer (pH 6.0) and boiled for 10 min in microwave oven. Non-specific binding was blocked by 5% BSA in PBS for 30 min. The slides were incubated with a 1∶300 dilution of antibody against human IL-17A (R&D Systems, Minneapolis, MN) at 4°C overnight in a moist chamber. Diaminobenzidine tetrahydrochloride was used as the visualization substrate followed by counterstaining with hematoxylin. Positively stained cells were counted under microscope by two independent investigators.

### Cell growth assay and focus formation assay

Cell growth rate was determined by XTT assay. Briefly, cells were seeded in a 96-well plate at a density of 1×10^3^ cells and incubated at 37°C in a humidified atmosphere containing 5% CO_2_. After 24 hr, cultured cells were treated with or without 50 ng/mL recombinant human IL-17A (rhIL-17A) (R&D System, Minneapolis, MN), and 10 ng/mL TNF-α (R&D System, Minneapolis, MN)was used as positive control. XTT assay using Cell Proliferation Kit II (Roche Molecular Biochemicals, Germany) was performed according to the manufacturer's instructions. Triplicate independent experiments were done and data were expressed as mean±SD. For focus formation assay, 1×10^3^ cells were seeded onto a 6-well plate and stimulated with or without 50 ng/ml rhIL-17A for 1 week. Surviving colonies were fixed and stained with 1% crystal violet. Triplicate independent experiments were performed.

### Cell migration and invasion assay

Cell migration and invasion ability were studied by wound healing and invasion assays. A series concentration of rhIL-17A (10 ng/ml, 50 ng/ml and 100 ng/ml) was tested and the result showed that 50 ng/ml rhIL-17A had the best effect (data did not shown), so 50 ng/ml rhIL-17A was used in this study. Cell migration was assessed by a scratch wound-healing assay. Cells were cultured in 6-well plate until confluent and then treated with or without rhIL-17A (50 ng/mL). The cell layer was wounded using a sterile tip and the spread of wound closure was observed and photographed under a microscope until healed area was found. Invasion assay was performed with 24-well BioCoat Matrigel Invasion Chambers (Becton Dicknson, Bedford, MA) according to the manufacturer's instructions. After cultured in medium with or without rhIL-17A (50 ng/mL), 5×10^4^ cells were seeded onto inner well and number of cells that invaded through the Matrigel was counted under 20× objective lens.

### Western Blot Analysis

Whole cell lysates from HCC cells were harvested with cell lysis buffer. Nuclear lysates from cultured PLC8024 and MHCC-97L cells were harvested with NucBuster™ ProteinExtraction Kit (Novagen, Germany) according to manufacturer's instructions. Western blotting analyses were performed with the standard protocol using antibodies against β-actin, E-cadherin, α-SMA, vimentin, P-p65 (Santa Cruz Biotechnology, Santa Cruz, CA), histone H3, fibronectin and MMP9, (Abcam,UK), N-cadherin, α-catenin and β-catenin (Cell Signalling Technology, Beverly, MA).

### Quantitative real-time PCR (qPCR)

Total RNA was extracted using TRIzol Reagent (Invitrogen, Carlsbad, CA), and reverse transcription was performed using an Advantage® RT for PCR Kit (Clontech, Mountain View, CA) according the manufacturer's instructions. For qPCR analysis, aliquot of double-stranded cDNA was amplified with primers (Table S1) using a SYBR Green PCR Kit (Applied Biosystems, Carlsbad, CA) and an ABI PRISM 7900 Sequence Detector. 18s rRNA was used as internal control. The threshold cycle (C_T_) was measured during the exponential amplification phase, and the amplification plots were analyzed using SDS 1.9.1 software (Applied Biosystems). The relative expression level of target genes (IL17A, MMP2 and MMP9) is given by 2^−ΔΔCT^ (ΔC_T_ = ΔC_T_^target^−ΔC_T_^18S^, ΔΔC_T_ = ΔC_T_^(target gene)^−ΔC_T_^(average of target gene in non-tumor tissue)^). All reactions were performed in duplicate.

### Statistical analysis

All data were analyzed with SPSS software (version 16.0) for statistical analysis. Comparisons between groups were analyzed by Student's t-test. Correlations between variables were determined by linear regression analysis. Survival was estimated by the Kaplan–Meier method and compared by the log-rank test. Univatiate and multivariate analysis of prognostic factor was performed with Cox progression model. Value of *P*<0.05 (two-tailed) was considered statistically significant.

## Supporting Information

Figure S1 **Inhibiting NF-κB with helenalin could block rhIL-17A induced HCC cell lines invasion.** Effect of NF-κB inhibitor on the blocking of rhIL-17A induced cell invasion was detected by cell invasive assay. Representatives of cells migrated through Matrigel-coated transwell were shown in the upper panel (magnification 100). Total invasive cell number in each chamber was summarized in the lower panel. *, *P*<0.05.
(TIF)

Table S1 **Primer list for qPCR.**
(DOC)
