# Supplementary figures and images for: Interleukin 17A Promotes Hepatocellular Carcinoma Metastasis via NF-kB Induced Matrix Metalloproteinases 2 and 9 Expression
Source: PLoS One. 2011 Jul 7;6(7):e21816. doi: 10.1371/journal.pone.0021816 (PMC3131399; doi:10.1371/journal.pone.0021816)

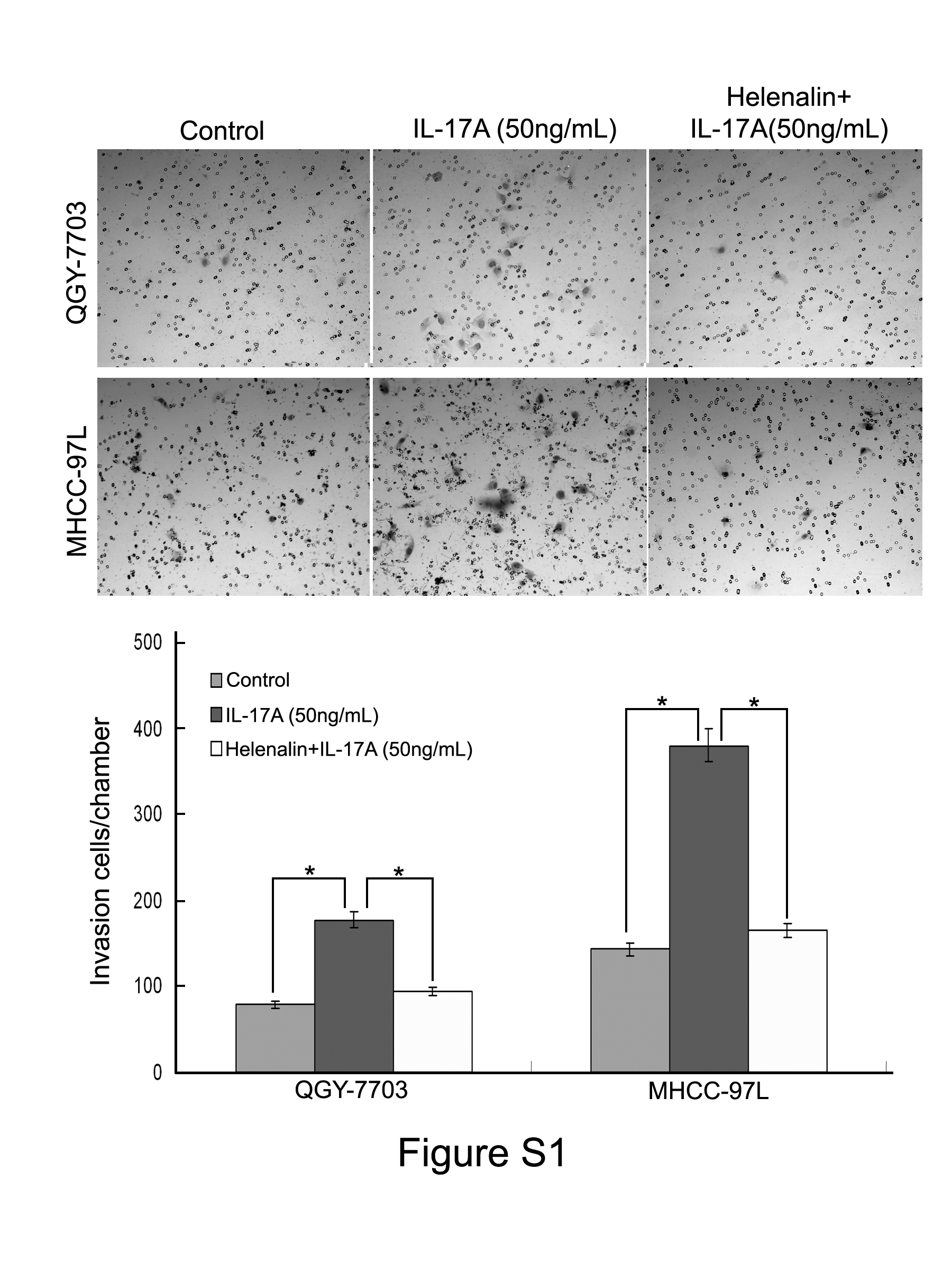

Supplement: Figure S1 — Inhibiting NF-κB with helenalin could block rhIL-17A induced HCC cell lines invasion. Effect of NF-κB inhibitor on the blocking of rhIL-17A induced cell invasion was detected by cell invasive assay. Representatives of cells migrated through Matrigel-coated transwell were shown in the upper panel (magnification 100). Total invasive cell number in each chamber was summarized in the lower panel. *, P<0.05. (TIF) [file pone.0021816.s001.tif]
