# Supplementary material for: Interleukin 17A Promotes Hepatocellular Carcinoma Metastasis via NF-kB Induced Matrix Metalloproteinases 2 and 9 Expression
Source: PLoS One. 2011 Jul 7;6(7):e21816. doi: 10.1371/journal.pone.0021816 (PMC3131399; doi:10.1371/journal.pone.0021816)
Supplement: Table S1 — Primer list for qPCR. (DOC) [file pone.0021816.s002.doc]

**Table S1: Primer list for qPCR**

| **ID** | **Sequence (5’-----3”)** |
| --- | --- |
| MMP1 forward primer | ACTCTGGAGTAATGTCACACCT |
| MMP1 reverse primer | GTTGGTCCACCTTTCATCTTCA |
| MMP2 forward primer | CCGTCGCCCATCATCAAGTT |
| MMP2 reverse primer | CTGTCTGGGGCAGTCCAAAG |
| MMP3 forward primer | AGTCTTCCAATCCTACTGTTGCT |
| MMP3 reverse primer | TCCCCGTCACCTCCAATCC |
| MMP9 forward primer | GGGACGCAGACATCGTCATC |
| MMP9 reverse primer | TCGTCATCGTCGAAATGGGC |
| MMP10 forward primer | CCCACTCTACAACTCATTCACAG |
| MMP10 reverse primer | TCAGATCCCGAAGGAACAGAT |
| IL-17A forward primer | CAATCCCACGAAATCCAGGATG |
| IL-17A reverse primer | GTGGAGATTCCAAGGTGAGG |
| 18S forward primer | CTCTTAGCTGAGTGTCCCGC |
| 18S reverse primer | CTGATCGTCTTCGAACCTCC |
